# Attraction of *Telenomus remus* to Egg Volatiles of *Spodoptera litura* and Oviposition‐Induced Plant Volatiles From Tobacco (*Nicotiana tabacum*)

**DOI:** 10.1002/ece3.72012

**Published:** 2025-09-18

**Authors:** Ibrahim Osman, Zhimin Wang, Hongnian Li, Ertao Li, Honglin Feng, Jiao Yin, Gemei Liang, Zhengling Liu, Dekai Ning, Kebin Li, Yonghui Xie

**Affiliations:** ^1^ State Key Laboratory for Biology of Plant Diseases and Insect Pests, Institute of Plant Protection Chinese Academy of Agricultural Sciences Beijing China; ^2^ Department of Entomology Louisiana State University Agricultural Center Baton Rouge Louisiana USA; ^3^ Kunming Branch of Yunnan Provincial Tobacco Company Kunming China

**Keywords:** *Spodoptera litura*, synthetic chemicals, *Telenomus remus*, tobacco, VOCs, Y‐tube olfactometer

## Abstract

The tobacco cutworm, *Spodoptera litura* F. (Lepidoptera: Noctuidae), is a polyphagous pest that causes enormous losses in tobacco production as it develops resistance to pesticides in China. *Telenomus remus* N. (Hymenoptera: Scelionidae) is an effective egg parasitoid for the genus Spodoptera. However, little is known about the volatile compounds that attract the parasitoid. In the present study, we investigated the response of *T. remus* to volatiles released from various sources, including *Spodoptera litura* egg masses, healthy tobacco plants, plants with manually introduced eggs, and plants with natural oviposition. The chemical profiles of the crude extracts were identified using gas chromatography–mass spectrometry (GC–MS), and the behavioral assay was performed using a Y‐tube olfactometer. Naïve and experienced *T. remus* showed significant attraction to crude extracts of *S. litura* egg masses and to oviposition‐induced plant volatiles (OIPVs). In subsequent experiments, different concentrations and combinations of the synthetic volatiles obtained from these crude extracts were tested. Notably, the binary and ternary mixtures showed significant attraction, where the binary combination of linalool (L) with tetracosane (T) and linalool (L) with cis‐3‐hexenyl acetate (C) was strongly attractive. These results underscore the potential of using synthetic volatile dispensers, particularly binary combinations with precise doses, to enhance retention of *T. remus* and improve the efficacy of the biological control program.

## Graphical abstract

The parasitoid *T. remus* was attracted to OIPVs and *S. litura* egg volatiles. At lower concentrations of 1 and 10 ng/μL the parasitoid showed the highest attraction. The binary combinations of linalool (L) with tetracosane (T) and linalool (L) with cis‐3‐hexenyl acetate (C) strongly attracted the parasitoids.

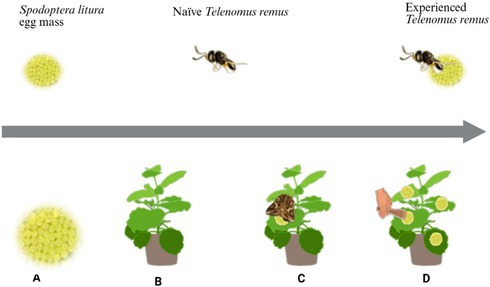

## 1 Introduction

Parasitoids utilize volatile organic compounds (VOCs) to locate their food sources and oviposition sites and to escape from their natural enemies (Van Oudenhove et al. 2017). These VOCs are primarily herbivore‐induced plant volatiles (HIPVs), which are released in response to herbivore attack (Veyrat et al. 2016). Plants use HIPVs as an indirect defense by attracting natural enemies of the attacking herbivores (Dicke et al. 2009; Martorana et al. 2017). HIPVs can also serve as host‐finding signals for egg parasitoids (Peñaflor et al. 2011). Egg parasitoids often use oviposition‐induced plant volatiles (OIPVs) as a reliable cue to locate their inconspicuous host (Moujahed et al. 2014; Hilker and Meiners 2010). Parasitoids can also use kairomones released by their host eggs when they are close to their host (Colazza et al. 2014; Tian et al. 2025). They can distinguish signals from host and non‐host species in multi‐trophic interaction (Cusumano et al. 2015; Wei Jianing et al. 2007). However, non‐host cues can sometimes mask specific host information (Zhang et al. 2009; Croijmans et al. 2022). Only a few compounds have ecological relevance in the complex blend of chemicals released into the environment. Therefore, identifying the specific compounds that attract the herbivore's natural enemy is paramount in mediating tri‐trophic interaction (Morawo and Fadamiro 2016; De Bruyne and Baker 2008). The use of synthetic volatiles can enhance the attraction of parasitoids. Several studies have shown that combinations of compounds are more effective in attracting parasitoids as they provide a more natural context than single compounds (Bargmann 2006). As reported (Van Neerbos et al. 2023), the aphid parasitoid *Aphidius colemani* V. (Hymenoptera: Braconidae) was more attracted to binary combinations of styrene and benzaldehyde in the greenhouse. Similarly, the specialist larval parasitoid *Microplitis croceipes* C. (Hymenoptera: Braconidae) was more attracted to an equivalent mixture of (Z)‐3‐hexanol and α‐pinene than to single compounds (Morawo and Fadamiro 2014).

Volatile organic compounds (VOCs), especially synthetic ones, are sustainable and environmentally friendly alternatives to conventional chemical pesticides. These semiochemicals play a key role in biological pest control by mimicking natural plant signals to attract natural enemies such as parasitoids and predators, thus increasing their effectiveness in agroecosystems (Turlings and Erb 2018). Due to their high specificity, VOCs can modulate certain insect behaviors such as host localization and oviposition, minimizing unintended effects on non‐target organisms and preserving ecological integrity. Their efficacy at low application rates helps to minimize pesticide use, thereby supporting food safety and environmental sustainability. In addition, mixtures of synthetic VOCs are more attractive to parasitoids than single compounds, as they better mimic the complexity of natural odor profiles (Van Neerbos et al. 2023). In addition to attracting insects, VOCs can also induce systemic acquired resistance in the emitter plant as well as in neighboring plants, providing an additional layer of defenses against pests and pathogens (Bouwmeester et al. 2019; Shi et al. 2024).

Egg parasitoids are key agents in biological pest control, targeting herbivorous insect eggs and thereby preventing damage by subsequent larval stages (Fatouros et al. 2016). Their ability to disrupt the development of pests at an early stage makes them particularly valuable for integrated pest management (IPM) strategies. To find suitable oviposition sites, these parasitoids rely on a range of sensory cues, including visual, plant‐emitted volatiles, and host‐derived chemical signatures (Park et al. 2025; Colazza et al. 2014; Manzano et al. 2022). In addition to airborne cues, contact chemoreception also plays a crucial role, as parasitoids can detect cuticular hydrocarbon residues left on plant surfaces by herbivores during feeding or locomotion. These chemical traces act as contact kairomones that provide reliable information about the presence of the host and increase the efficacy of host search (Wäschke et al. 2013; Tognon et al. 2020).

The tobacco caterpillar, *Spodoptera litura* F. (Lepidoptera: Noctuidae), is a highly destructive and polyphagous pest that attacks a wide range of economically important crops, such as tobacco, cotton, maize, soybean, and peanut (Saraswathi et al. 2023). The larvae attack plants from the seedling to early reproductive stages (Natikar and Balikai 2017). Recurrent infestations have led to crop losses of 25% to 100% in various agricultural regions (Shekhawat et al. 2018; Shilpa and Remia 2017). The intensive and prolonged use of synthetic insecticides has led to widespread resistance to several chemical classes, including pyrethroids, organophosphates, carbamates, and organochlorines (Huang and Han 2007), undermining the effectiveness of conventional pest control strategies.

Telenomus *remus* (Nixon 1973) (Hymenoptera: Scelionidae) is an effective egg parasitoid of lepidopteran pests, especially species in the genus Spodoptera. Although it was originally described as a parasitoid of *Spodoptera frugiperda* S. (Lepidoptera: Noctuidae), *S. litura* has been proven to be a more suitable host for mass‐rearing (Chen, Li, et al. 2021). Its high reproductive capacity, female‐biased sex ratio, resistance to certain insecticides, and ease of rearing in the laboratory make *T. remus* a promising candidate for IPM programs (Chen, Weng, et al. 2021; Agboyi et al. 2021). The use of *T. remus* in applied biological control is well documented (Shen et al. 2023; Chen et al. 2023). Field studies in Brazil have reported parasitism rates of *T. remus* on *S. frugiperda* eggs ranging from 54% to 99% in crops such as maize, cotton, and soybean (Pomari et al. 2013). Despite its high parasitization potential on *S. litura* eggs, *T. remus* is not used in tobacco agroecosystems, where *S. litura* continues to cause significant economic damage annually (Rao et al. 2004). This limited use is largely due to a lack of detailed knowledge of the volatile organic compounds (VOCs) that influence the parasitoids' host‐searching behavior.

While numerous studies have demonstrated the role of HIPVs and OIPVs in parasitoid attraction (Nurkomar et al. 2018, 2017), there is little evidence for the attraction of *T. remus* by volatiles emitted from *S. litura* ‐infested tobacco plants. Therefore, the aim of the present study was to investigate the semiochemical cues that influence the behavioral response of *T. remus* to *S. litura* eggs, undamaged tobacco plants, natural oviposition of *S. litura* on tobacco plants, and tobacco plants inoculated with *S. litura* eggs. To determine the most effective attractants, we evaluated the behavioral responses of *T. remus* to individual synthetic VOCs and their binary and ternary combinations at different concentrations using Y‐tube olfactometer bioassays.

## 2 Materials and Methods

### 2.1 Plant Treatments

**FIGURE 1:**
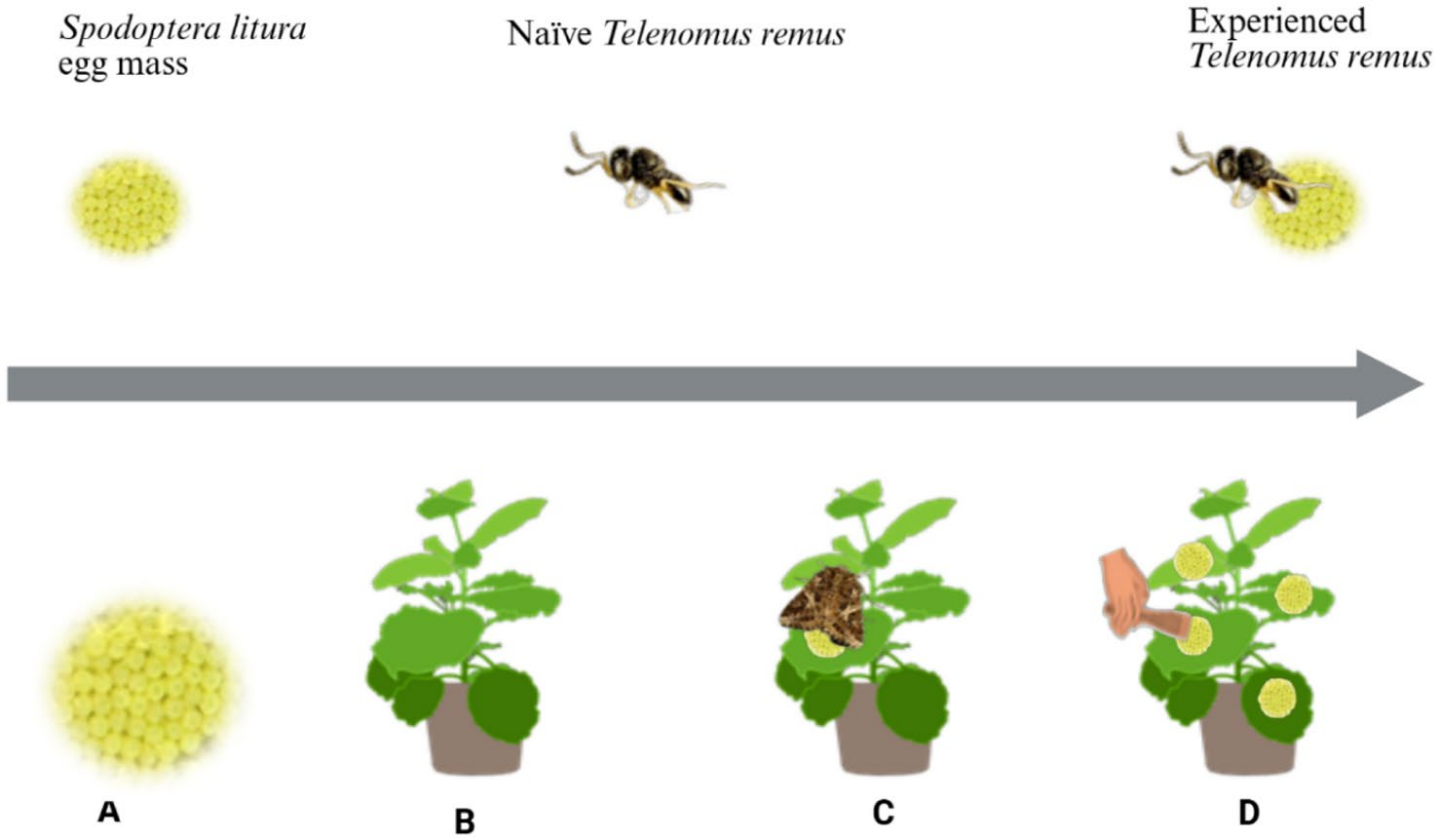
Treatments set‐up used in the experiments as sources of volatiles: (A) *S. litura* egg masses, (B) Healthy tobacco plant, (C) Tobacco plant with naturally ovipositing *S. litura* , (D) Tobacco plants with manually introduced *S. litura* egg masses.

The seeds of tobacco plants ( *Nicotiana tabacum* ) were procured from Yuxi Zhong Yan Tobacco Seed Co., Ltd. Then, the seeds were sown in cylindrical plastic pots (10 cm in diameter) three‐quarters filled with a mixture of soil and fertilizer. The fertilizer was from Heilongjiang Wuchang Luduo Seedling Substrate Co. Ltd. The plants were cultivated under standard laboratory conditions (approximately 25°C ± 2°C, 50%–70% relative humidity, and a 14:10 light: dark photoperiod). Seedlings were used for the experiment when they were 10 cm tall. The plant treatments were prepared as follows: (a) Healthy plants: Tobacco plants grown under controlled environmental conditions were used without prior exposure to herbivores; served as baseline controls for volatiles emitted from the plants. (b) Natural oviposition: Thirty adult *S. litura* moths (20 females and 10 males) were introduced into insect‐rearing cages containing undamaged tobacco plants and allowed to lay eggs. After oviposition, the plants were left undisturbed for 36, 60, and 84 h before volatile collection to capture temporal changes in plant responses. (c) Artificially inoculated eggs: Undamaged tobacco plants were manually inoculated with *S. litura* eggs. Volatile collections were performed at 24, 48, and 72 h after inoculation to assess plant responses at different stages of egg presence. To avoid contamination of volatile plant profiles by compounds extracted from the eggs, egg masses were carefully removed with a fine brush during both natural oviposition and artificial inoculation prior to the collection of volatile samples. The treatment setups for the extraction of crude volatiles are shown in Figure 1.

### 2.2 Insect Rearing

The egg masses of *S. litura* were collected from the Institute of Plant Protection, CAAS, Beijing, and kept in plastic bags at 25°C ± 1°C until hatching. The newly emerged larvae were fed with an artificial diet in a large plastic box (28 × 9 × 9 cm) as reported (Gupta et al. 2005) until they reached the fifth instar. The fifth instar larvae were then transferred to a similarly sized plastic box with moist soil to prevent desiccation and facilitate pupation. Adult moths were reared in a cylindrical wire‐mesh cage (28 cm height with 24 cm diameter) at 25°C ± 1°C and fed with a 10% honey solution.

Parasitoid *T. remus* was obtained from Beijing Zhihui Chong Biotechnology Co. Ltd., China, and reared in a climate chamber at 26°C ± 1°C, 70% ± 5% relative humidity, and a 14:10 (light:dark) photoperiod (Chen et al. 2023). Newly emerged adults were provided with a 10% honey solution. After 4 h, the parasitoids were anesthetized with carbon dioxide for 1 min, and the sexes were separated based on the antennal morphology. Crude volatile extracts from the four treatments were tested on both naïve and experienced *T. remus* females to compare innate and learned odor responses. Naïve females were not exposed to *S. litura* eggs, while experienced females were exposed to the eggs for 24 h prior to the assays. Synthetic VOCs, including individual compounds and blends, were evaluated exclusively with naïve females. All experiments using the Y‐tube olfactometer were performed with 24–48‐h‐old females to ensure behavioral consistency.

### 2.3 Headspace Volatile Collection and GC–MS Analysis

**FIGURE 2:**
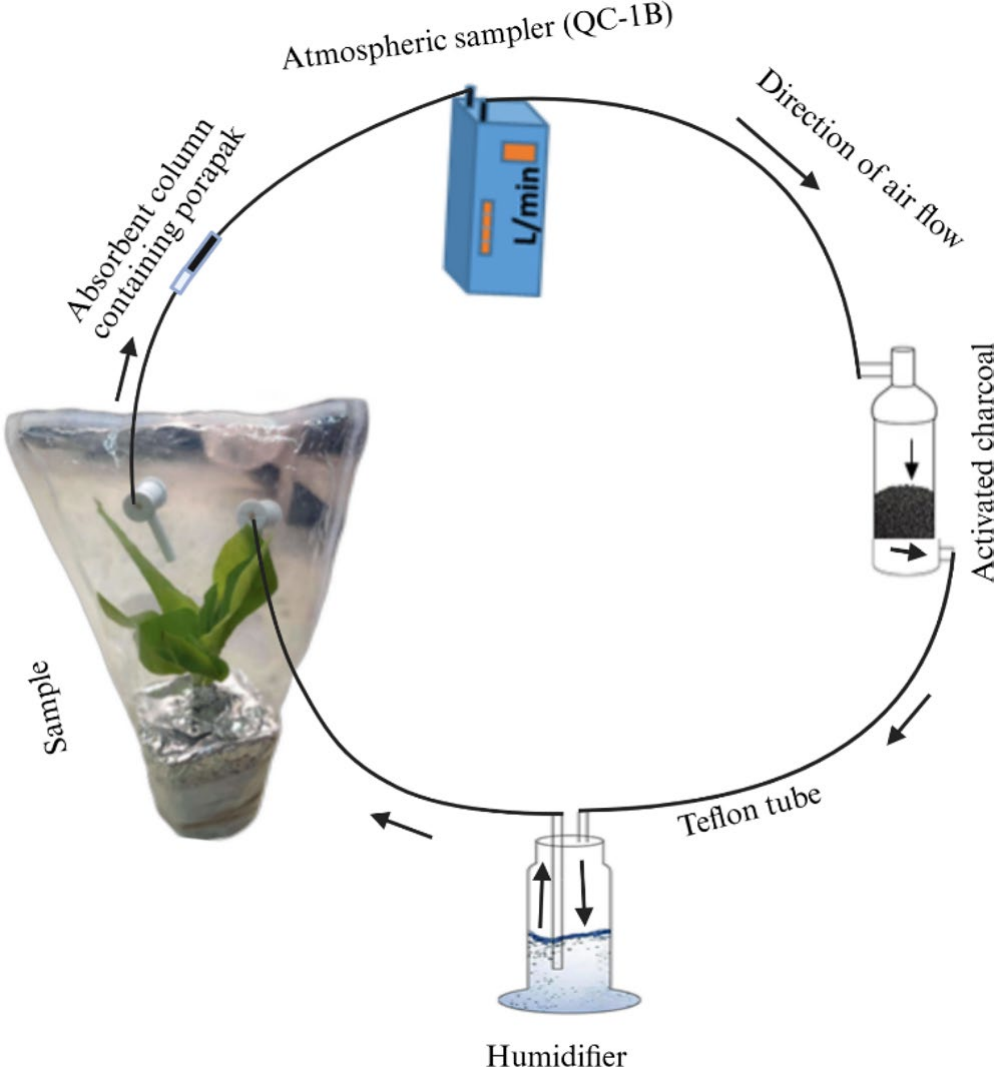
Schematic representation of dynamic headspace volatile collection.

A dynamic headspace collection system was used to collect volatiles from *S. litura* eggs, tobacco plants with natural oviposition, tobacco plants inoculated with eggs, and undamaged tobacco plants (Figure 2). According to the timing in previous studies (Fatouros et al. 2012; Cusumano et al. 2015), plant volatiles from natural oviposition and manually introduced egg masses were collected 36, 60, and 84 h after egg laying and 24, 48, and 72 h after manual introduction of the eggs. The Teflon tubes and the bag used to cover the plant were first sterilized with alcohol and placed in a hot oven at a temperature of 60°C for 1 h. Then, all Y‐tube components were connected overnight to clean the chemicals released from the empty plastics. The next day, the plants were connected and left for 2 h before the column containing Porapak was connected to avoid unnecessary contamination. During volatile collection, the plants covered with the polyethene bag were sealed airtight at the center of the pot (Figure 2). The airflow from the roots was blocked by aluminum foil wrapped around the top of the container. The air flows in a closed system pumped by an atmospheric sampler (QC‐1B) at a rate of 200 mL/min. An adsorbent column containing Porapak Q (20 mg, 80–100 mesh; CNW Technologies, Düsseldorf, Germany) was attached to the inlet. After 10 h of continuous collection, the volatiles were eluted from the Porapak Q filter with 1 mL of hexane. The extracted solution was stored at −20°C until the bioassay test. The procedure for collecting the volatiles from the *S. litura* eggs was the same as for the plant treatment. 150 g of eggs were used for each collection. The crude extracts from each treatment were analyzed using a Shimadzu single quadrupole gas chromatograph coupled with mass spectrometry (GC–MS‐QP2010 SE, SKL‐0108) equipped with an HP‐5MS UI column (Agilent, USA; 30 m long × 0.25 mm inner diameter × 0.25 μm film thickness). A sample of 1 μL aliquot of each crude extract was injected into a polar capillary column (HP‐1 MS, 30 m, 0.25 mm ID, 0.25 μm film thickness; Agilent J&W Scientific, USA) in pulsed splitless mode. Helium was used as the carrier gas. After injection, the column temperature was first held at 40°C for 1 min and then increased to 200°C at 1°C/min, followed by a post‐run at 280°C for 10 min. The chemical profile of the synthetic chemicals was identified using the NIST17 online libraries.

### 2.4 Y‐Tube Olfactometer Assay

Y‐tube olfactometer assays were used to evaluate the behavioral responses of both naïve and experienced *T. remus* females to crude volatile extracts from egg masses of *S. litura* alone (SEVs), manually introduced *S. litura* eggs‐induced plant volatiles (MEIPVs), adult *S. litura* females laying eggs on the plant (OIPVs), and healthy tobacco plants (HPVs). In contrast, the behavioral assays involving synthetic compounds identified by GC–MS analysis were performed exclusively using naïve parasitoids. This decision was based on previous observations indicating that there were no significant behavioral differences between naïve and experienced females in response to crude volatile extracts. The main target was to identify the chemical compounds that influence the behavior of *T*. *remus* from the four treatments. After we conducted the behavioral test for the crude extracts, the parasitoid showed significant attraction to OIPVs and *S. litura* egg volatiles in a Y‐tube olfactometer. Furthermore, the individual chemicals identified by the gas chromatograph coupled with mass spectrometry (GC–MS) were also assessed in different concentrations and combinations.

The glass tube olfactometer consisted of a main tube measuring 10 cm in length and 2 cm in inner diameter, branching into two arms of equal length (10 cm) positioned at a 60° angle. Each arm was connected to a 5 cm long glass adapter with a 1 cm inner diameter. All parts of the Y‐tube setup were interconnected with Teflon tubes. To ensure clean airflow, the air from the external environment was purified with activated charcoal and subsequently humidified by passing it through a tap water bottle. The flow rate at each arm was set at 500 mL/min. A fluorescent lamp was placed at the top center of the stainless‐steel light chamber (100 × 80 × 80 cm) to ensure uniform light distribution.

**FIGURE 3:**
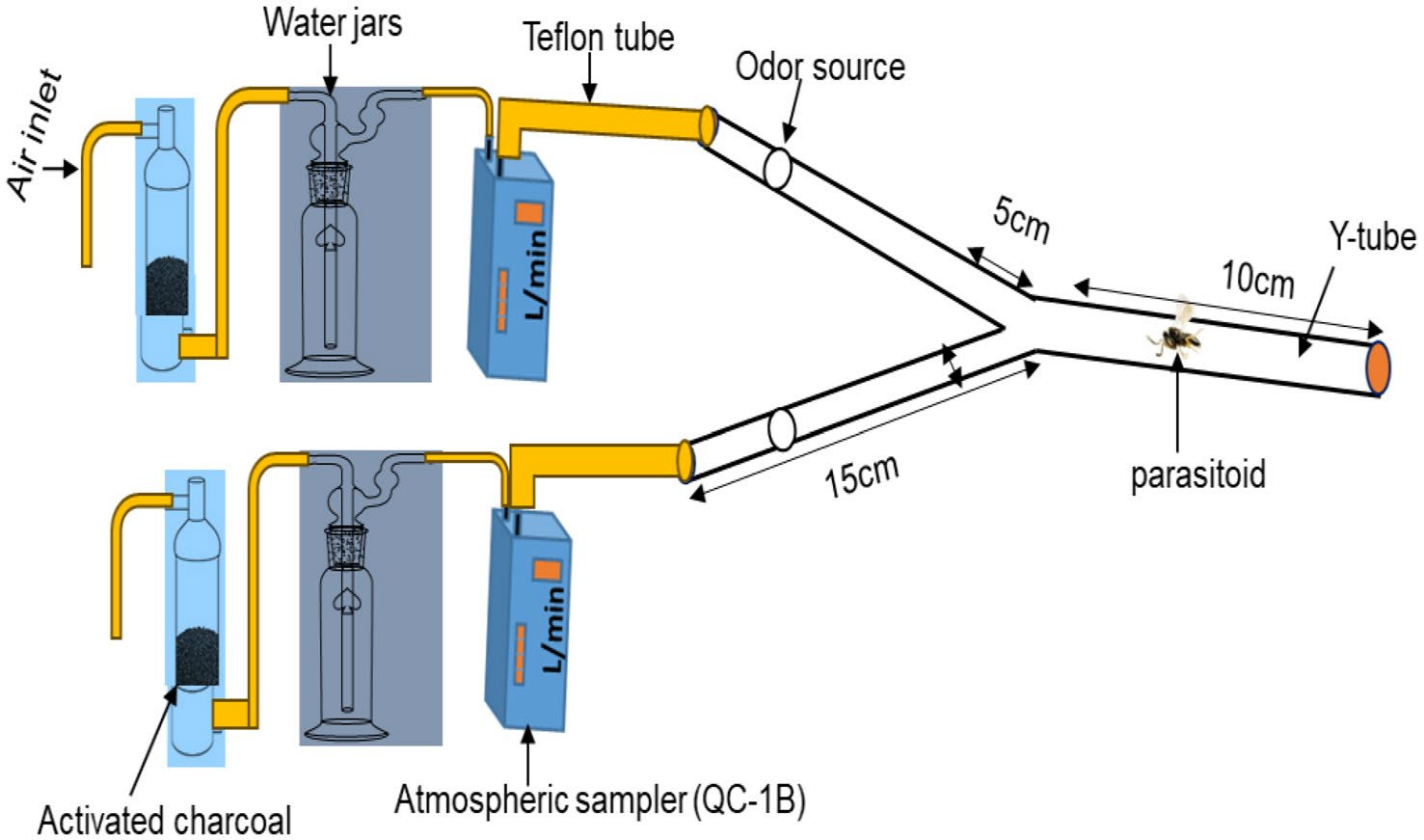
Schematic diagram of the Y‐tube olfactometer used to evaluate the behavior of the parasitoid *T. remus*.

Each crude extract was eluted from the Porapak Q with 1 mL of hexane. The synthetic compounds were diluted in hexane to the desired concentrations (1, 10, 100, and 100 ng/μL). For each assay, 10 μL of the test solution was applied to a 2 × 2 cm piece of filter paper to ensure a sufficient mass of the volatile compound in the sample. The filter paper was immediately placed on one arm of the olfactometer, while a filter paper with a control was placed on the opposite arm. For crude extract assays, healthy plant volatiles served as the control, whereas for synthetic compound hexane alone was used as the control. The stimuli were delivered to the olfactometer arms at a constant airflow rate of 500 mL/min. The parasitoids were introduced into the Y‐tube individually, and the test chamber was closed after the introduction of the parasitoid; 5 min later, the chamber was opened, and the parasitoid was recorded as a choice or no choice based on the position indicated on the Y‐tube (Figure 3). If the parasitoids crossed the line on the arm approximately 5 cm from the intersection of the two arms, it was scored as a choice; otherwise, it was marked as a non‐responder. The position of the arm was changed after five parasitoids to avoid position bias. After testing 15 parasitoids, all the instruments were substituted with new ones. In each test, 90 parasitoids were used, and each female was considered a replicate. At the end of the experiment, all the equipment was washed with distilled water and cleaned with 75% ethanol, and then all glass equipment was dried at 100°C for 30 min to minimize the effects of residual odor for the next use. The experiments were conducted during the daytime (10 am to 5 pm). Wasps that did not make a choice were excluded from the analysis.

### 2.5 Synthetic Volatile Chemicals

A total of 26 chemicals were selected from the chemicals identified by Gas chromatography mass spectrometry (GC–MS) for Y‐tube olfactometer assays based on their ecological relevance, literature support, and availability. It has previously been reported that (Z)‐9‐dodecen‐1‐ol acetate (Z‐9‐DDA), a kairomone from the abdominal tips of *S. frugiperda* females, attracts *T. remus* (Nordlund et al. 1983) and chemicals such as linalool and indole released from undamaged maize plants also elicited a behavioral response in the parasitoid (Michereff et al. 2019). These results support research on chemosensory responses of *T. remus* for targeted pest control. In the current study, each chemical was initially tested at a concentration of 10 ng/μL, a dosage considered moderate and suitable for preliminary screening, as it can elicit electrophysiological responses without causing a repellent effect to the parasitoids (Ali et al. 2022).

The compounds that showed significant attraction to the parasitoid in the initial screening were then tested at four serially diluted concentrations (1, 10, 100 and 1000 ng/μL), following the protocols of previous studies (Devescovi et al. 2024; Yi et al. 2023; Liu et al. 2024), to determine the concentration that elicited the strongest behavioral response. The concentrations that showed higher attraction for each compound tested were further used in the formulation of binary and ternary combinations. The optimal concentrations used in the binary and ternary combinations were 1 ng/μL for linalool and heneicosane and 10 ng/μL for the remaining compounds. Since plants naturally emit volatile organic compounds as complex blends, it is essential to simulate this ecological context in order to effectively attract natural enemies of herbivores. Therefore, the concentration that elicited the highest parasitoid attraction in individual assays was considered the optimal concentration and subsequently used in the formulation of binary and ternary mixtures. Morawo and Fadamiro (2016) reported that a 1 μg/μL concentration of synthetic chemicals was used as optimal for binary combination. The binary compounds were formulated in a 1:1 ratio, while the ternary mixtures were formulated at a ratio of 2:1:1. A chemical sample of 10 μL of the mixture was loaded on a filter paper (2 × 2 cm), which was then placed in the treatment arm of the Y‐tube. The same amount of hexane was placed in the other arm as a control. Hexane was used as a solvent for all chemicals and as a control for the bioassay. The binary and ternary combinations used in this experiment are given in Table 1.

**TABLE 1:** Binary and ternary mixtures of chemicals used in the Y‐tube bioassay.

Blends	Binary combinations 1:1	Ternary combinations 2:1:1
1	C + H	L + D + C
2	H + P	L + D + H
3	D + C	L + D + P
4	C + P	L + D + T
5	L + C	L + C + H
6	D + T	L + C + P
7	P + T	L + C + T
8	L + T	L + H + P
9	C + T	L + H + T
10	L + P	L + P + T

Abbreviations: C, Cis‐3‐hexenyl acetate; D, Decanal; H, Heneicosane; L, Linalool; P, (+)‐α‐pinene; T, Tetracosane.

### 2.6 Statistical Analyses

All analyses were performed using SAS 9.2 with a 0.05 level of significance. Data are presented as mean ± SE. The responses of parasitoids between treatments and controls were analyzed using the Chi‐square test (*χ* ^2^). Wasps that did not cross the choice line were deemed no‐choice and excluded from the analyses. The graphs were generated using GraphPad Prism v8.0 (San Diego, CA, USA).

## 3 Results

### 3.1 Responses of *T. remus* to Crude Extracts in Y‐Tube Assay

**FIGURE 4:**
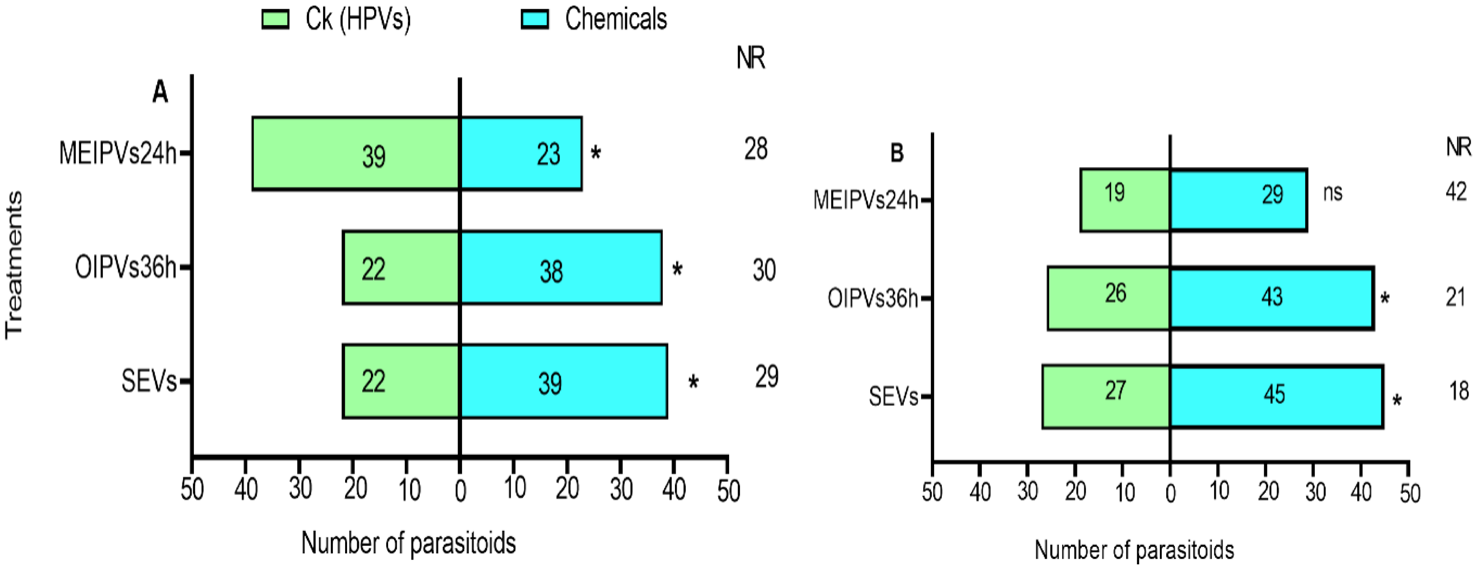
Responses of *T. remus* females to SEVs, MEIPVs, OIPVs, and HPVs (control) in a Y‐tube olfactometer. (A) Response of naïve parasitoids. (B) Response of experienced parasitoids. Bars represent (means ± SE); asterisk represents *p < 0.05, for *χ* ^2^ test; NR, non‐respondents; ns, not significant.

Both naïve and experienced *T. remus* were attracted to OIPVs and SEVs compared to HPVs (Figure 4). Specifically, naïve *T. remus* was significantly attracted to SEVs (*χ* ^2^ = 4.738; df = 1; *p* = 0.030) and OIPVs (*χ* ^2^ = 4.267; df = 1; *p* = 0.039) compared to the HPVs control. In addition, the parasitoids were more attracted to HPVs (*χ* ^2^ = 4.267; df = 1; *p* = 0.039) than to MEIPVs (Figure 4A). Experienced parasitoids were also significantly attracted to SEVs (*χ* ^2^ = 4.500; df = 1; *p* = 0.034) and OIPVs (*χ* ^2^ = 4.188; df = 1; *p* = 0.041) compared to the HPVs control. MEIPVs (*χ* ^2^ = 2.083; df = 1; *p* = 0.149) showed no difference in attraction to experienced parasitoids over HPVs (Figure 4B).

### 3.2 Timing of Volatile Collection in *T. remus* Attraction

**FIGURE 5:**
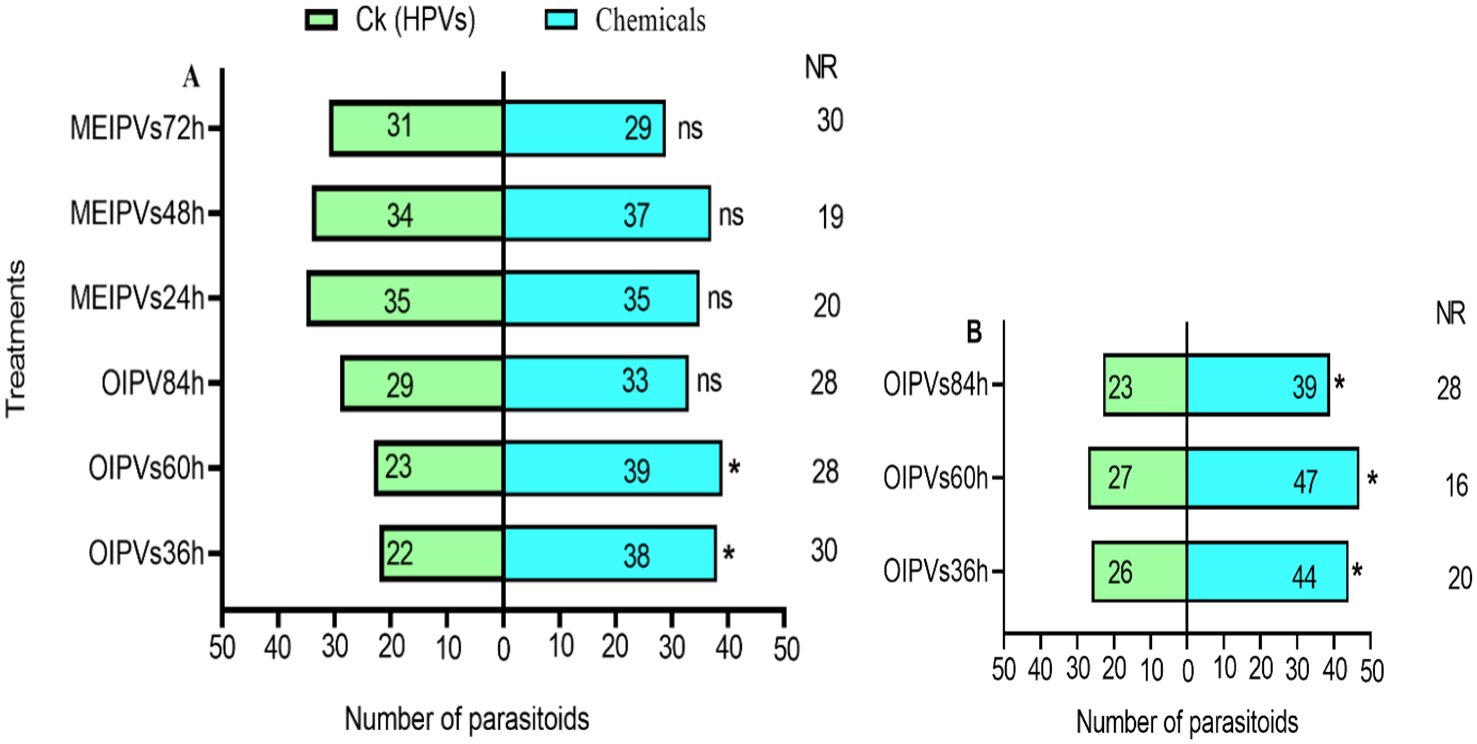
Response of *T. remus* females to MEIPVs after 24, 48, and 72 h, OIPVs after 36, 60, and 84 h, and the HPVs (control). (A) Response of naïve parasitoids. (B) Response of experienced parasitoids. Bars represent (means ± SE); asterisk represents *p < 0.05 for *χ* ^2^ test; NR, non‐respondents; ns, not significant.

The parasitoid response was further evaluated to volatiles collected at different time points (Figure 5). Naïve parasitoids were significantly attracted to OIPVs collected 36 h after oviposition (*χ* ^2^ = 4.267; df = 1; *p* = 0.039) and 60 h after oviposition (*χ* ^2^ = 4.129; df = 1; *p* = 0.042) compared to the HPVs control. However, the parasitoids showed no preference to OIPVs collected 84 h after oviposition (*χ* ^2^ = 0.258; df = 1; *p* = 0.611) (Figure 5A). Experienced parasitoids were also significantly attracted to OIPVs collected 36 h (*χ* ^2^ = 4.629, df = 1; *p* = 0.031), 60 h (*χ* ^2^ = 5.405; df = 1; *p* = 0.020), and 84 h (*χ* ^2^ = 4.129; df = 1; *p* = 0.042) after oviposition compared to the HPVs control (Figure 5B).

### 3.3 Identification of Volatiles Emitted From Healthy Plant, *S. litura* Eggs and OIPVs From Tobacco

Gas chromatography mass spectrometry (GC–MS) analysis identified 73 and 84 compounds in the crude extracts from the healthy plant and OIPVs, respectively, and 34 chemical compounds from *S. litura* eggs. Because MEIPVs did not show significant attraction to *T. remus* in the behavioral tests, the GC–MS result is not included. Therefore, 26 chemicals were selected from the chemicals identified in all treatments for Y‐tube olfactometer assays based on information in the literature, availability, and ecological relevance of the chemicals. All tested chemicals and the source where they were obtained are indicated in Table 2.

**TABLE 2:** Synthetic volatile chemicals used in the Y‐tube olfactometer and their respective treatment source.

S. No	Compound	MF	Treatment				
			SEVs	HPVs	OIPVs		
					36 h	60 h	84 h
1	Nonanal	C_9_H_18_O		+	+	+	+
2	Pentadecane	C_15_H_32_	+				
3	Undecane	C_11_H_24_	+				
4	Squalene	C_30_H_50_		+			
5	Eicosane	C_20_H_42_	+				
6	Heneicosane	C_21_H_44_	+				
7	Decanal	C_10_H_20_O		+	+	+	
8	Cis‐3‐Hexenyl Acetate	C_8_H_14_O_2_		+	+	+	+
9	(+)‐α‐pinene	C_10_H_16_			+	+	+
10	Dodecane	C_12_H_26_		+			
11	Nonadecane	C_19_H_40_	+				
12	Heptadecane	C_17_H_36_	+				
13	Octanal	C_8_H_16_O		+	+		
14	1‐Hexanol	C_6_H_14_O		+		+	
15	Ethyl Benzene	C_8_H_10_		+			
16	Tridecane	C_13_H_28_	+				
17	Octane	C_8_H_18_		+			
18	Tetracosane	C_24_H_50_	+				
19	Styrene	C_8_H_8_		+	+		
20	Hexadecane	C_16_H_34_	+				
21	Tetradecane	C_14_H_30_	+				
22	Linalool	C_10_H_18_O			+	+	+
23	Decane	C_10_H_22_		+	+		
24	3‐Hexanol	C_6_H_14_O			+		
25	Butyl butyrate	C_8_H_16_O_2_		+			
26	Nonane	C_9_H_20_			+	+	

*Note:* “+” represents the treatment where the compound obtained.

Abbreviations: HPVs, healthy plant volatiles; MF, molecular formula of the compound; OIPVs, oviposition‐induced plant volatiles; SEVs, *Spodoptera litura* egg volatiles.

Among the 26 chemical compounds tested, 11 influenced the behavioral response of *T. remus* (Table 3). Of these, six compounds showed a statistically significant attractive effect, whereas five elicited a repellent response. The attraction of *T. remus* to different concentrations of six synthetic compounds such as cis‐3‐hexenyl acetate, linalool, decanal, (+)‐α‐pinene, tetracosane and heneicosane was tested using a Y‐tube olfactometer. The 11 chemical compounds showed effect on *T. remus* behavior (Table 3).

**TABLE 3:** Volatiles collected from healthy plant, *S. litura* eggs, and OIPVs that showed attraction and repulsion effect on *T. remus*.

S. No	Compound	MF	Effect
1	1‐Hexanol	C_6_H_14_O	Repel
2	Hexadecane	C_16_H_34_	Repel
3	Cis‐3‐hexnyl acetate	C_8_H_14_O_2_	Attract
4	Decane	C_10_H_22_	Repel
5	(+)‐α‐pinene	C_10_H_16_	Attract
6	Decanal	C_10_H_20_O	Attract
7	Linalool	C_10_H_18_O	Attract
8	Pentadecane	C_15_H_32_	Repel
9	Nonadecane	C_19_H_40_	Repel
10	Heneicosane	C_21_H_44_	Attract
11	Tetracosane	C_24_H_50_	Attract

### 3.4 Parasitoid Response to Synthetic Chemicals at a Single Concentration

**FIGURE 6:**
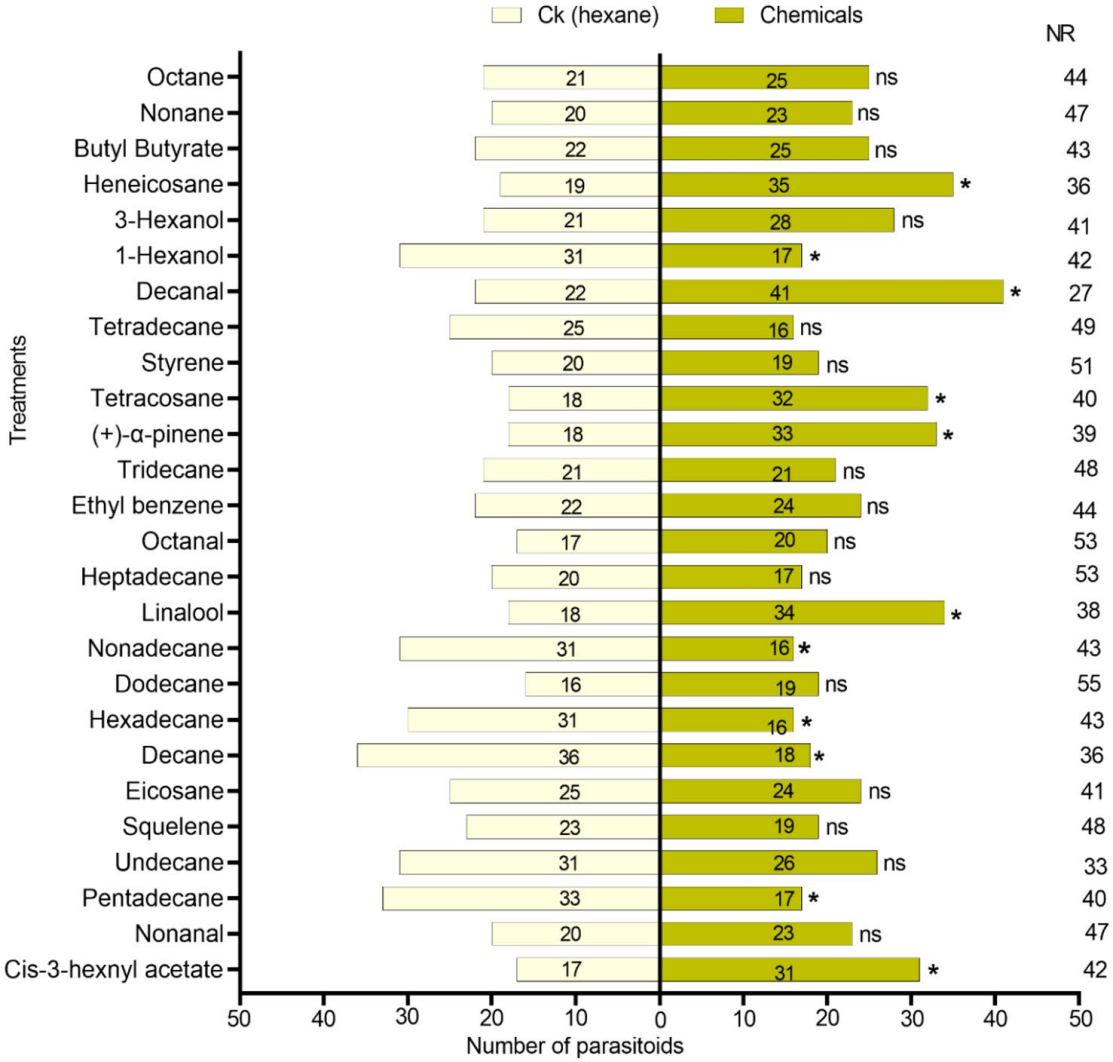
Response of *T. remus* females to synthetic chemicals obtained from the GC–MS. Bars represent means ± SE; asterisks represent *p < 0.05 under *χ* ^2^ tests, and NR, non‐respondents; ns, no significant.

The selected chemicals were tested at 10 ng/μL concentration for screening the effective chemicals. The chemical cis‐3‐hexenyl acetate showed significant attraction with a notable *p*‐value indicating strong statistical significance (*χ* ^2^ = 4.083; df = 1; *p* = 0.043), Linalool (*χ* ^2^ = 4.923; df = 1; *p* = 0.027), (+)‐α‐pinene (*χ* ^2^ = 4.412; df = 1; *p* = 0.036), tetracosane (*χ* ^2^ = 3.920; df = 1; *p* = 0.048), decanal (*χ* ^2^ = 5.730; df = 1; *p* = 0.017), and heneicosane (*χ* ^2^ = 4.741; df = 1; *p* = 0.029). Certain chemicals showed a repellent effect, and the parasitoid was attracted to the control (Figure 6). The parasitoid showed a repulsion response to pentadecane (*χ* ^2^ = 5.120; df = 1; *p* = 0.024), decane (*χ* ^2^ = 6.000; df = 1; *p* = 0.014), hexadecane (*χ* ^2^ = 4.261; df = 1; *p* = 0.039), nonadecane (*χ* ^2^ = 4.787; df = 1; *p* = 0.029) and 1‐hexanol (*χ* ^2^ = 4.083; df = 1; *p* = 0.043).

### 3.5 Parasitoid Response to Different Concentrations of Effective Chemicals

**FIGURE 7:**
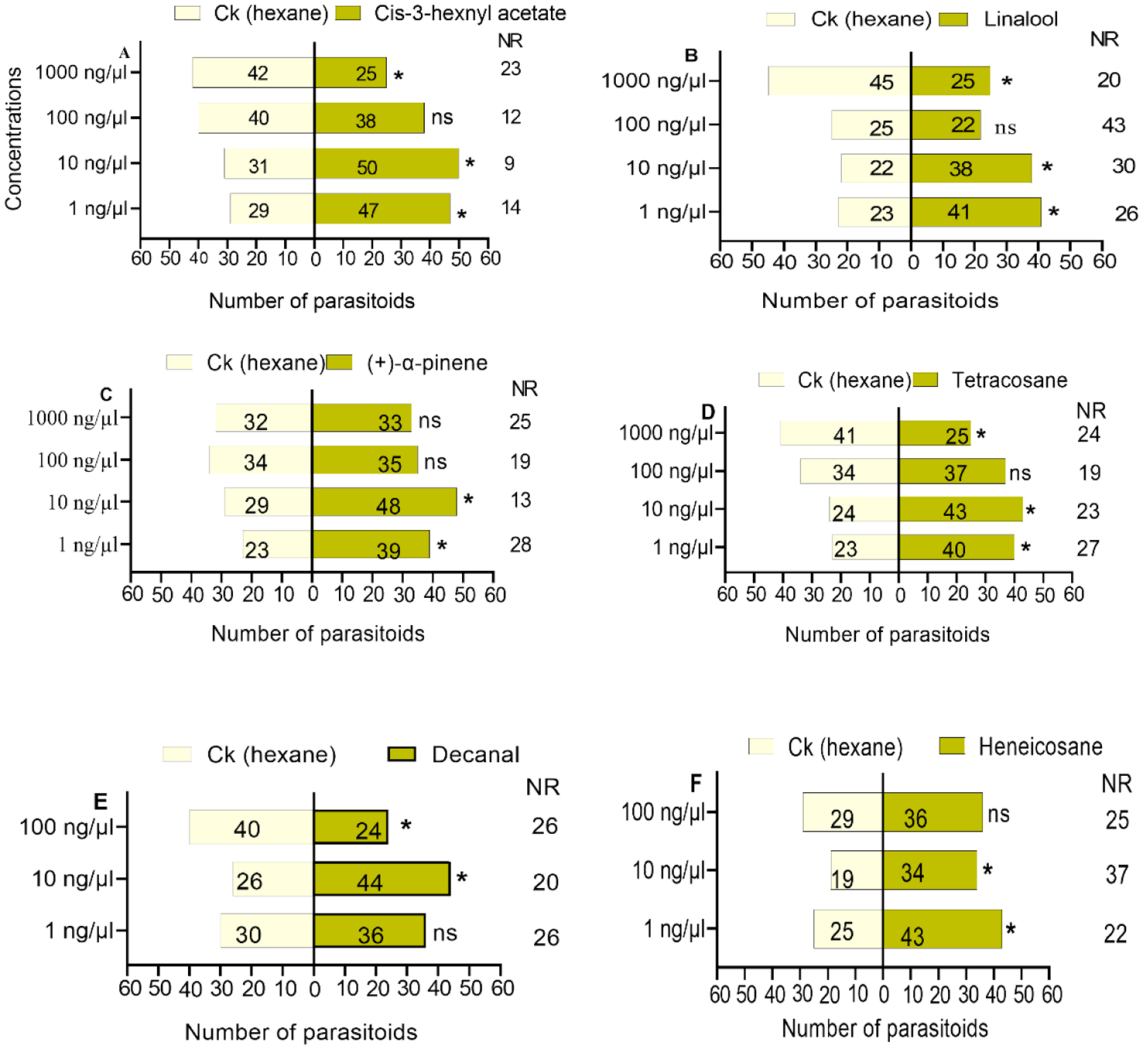
Response of *T. remus* females to different concentrations of the effective compounds. (A) Cis‐3‐hexenyl acetate, (B) Linalool, (C) (+)‐α‐pinene, (D) Tetracosane, (E) Decanal and (F) Heneicosane. Bars represent means ± SE; NR, non‐respondents; ns, no significance, and an asterisk represents *p < 0.05, *χ* ^2^ test.

The parasitoids exhibited notable attraction at the lower concentrations of 1 and 10 ng/μL across the majority of the six chemicals. Cis‐3‐hexenyl acetate (Figure 7A) showed significant attraction at 1 ng/μL (*χ* ^2^ = 4.263; df = 1; *p* = 0.039) and 10 ng/μL (*χ* ^2^ = 4.457; df = 1; *p* = 0.035). No significant attraction was observed at 100 ng/μL (*χ* ^2^ = 0.051; df = 1; *p* = 0.821), while repulsion occurred at a concentration of 1000 ng/μL (*χ* ^2^ = 4.313; df = 1; *p* = 0.038). Linalool (Figure 7B) showed higher attraction at a concentration of 1 ng/μL (*χ* ^2^ = 5.063; df = 1; *p* = 0.024) and 10 ng/μL (*χ* ^2^ = 4.267; df = 1; *p* = 0.039). At a concentration of 100 ng/μL, there was no significant difference observed (*χ* ^2^ = 0.191; df = 1; *p* = 0.662). However, at 1000 ng/μL, a repulsion effect was noted (*χ* ^2^ = 5.714; df = 1; *p* = 0.017). (+)‐α‐pinene (Figure 7C) showed significant attraction at 1 ng/μL (*χ* ^2^ = 4.129; df = 1; *p* = 0.042) and 10 ng/μL (*χ* ^2^ = 4.688; df = 1; *p* = 0.030). No significant attraction was observed at 100 ng/μL (*χ* ^2^ = 0.014; df = 1; *p* = 0.904) and 1000 ng/μL (*χ* ^2^ = 0.015; df = 1; *p* = 0.901). Tetracosane (Figure 7D) showed significant attraction at 1 ng/μL (*χ* ^2^ = 4.587; df = 1; *p* = 0.032) and 10 ng/μL (*χ* ^2^ = 5.388; df = 1; *p* = 0.020). The results indicated no significant attraction at a concentration of 100 ng/μL (*χ* ^2^ = 0.127; df = 1; *p* = 0.722). In contrast, the parasitoids showed attraction to control (hexane) at 1000 ng/μL (*χ* ^2^ = 3.879; df = 1; *p* = 0.049). Decanal (Figure 7E) showed significant attraction at 10 ng/μL (*χ* ^2^ = 4.629; df = 1; *p* = 0.031). No significant attraction was observed at 1 ng/μL (*χ* ^2^ = 0.545; df = 1; *p* = 0.460) but repulsion occurred at 100 ng/μL (*χ* ^2^ = 4.000; df = 1; *p* = 0.046). Heneicosane (Figure 7F) showed strong attraction at 1 ng/μL (*χ* ^2^ = 4.765; df = 1; *p* = 0.029) and 10 ng/μL (*χ* ^2^ = 4.245; df = 1; *p* = 0.039). No significant attraction was observed at 100 ng/μL (*χ* ^2^ = 0.754; df = 1; *p* = 0.385).

### 3.6 Parasitoid Response to Binary and Ternary Combinations of Effective Chemicals

**FIGURE 8:**
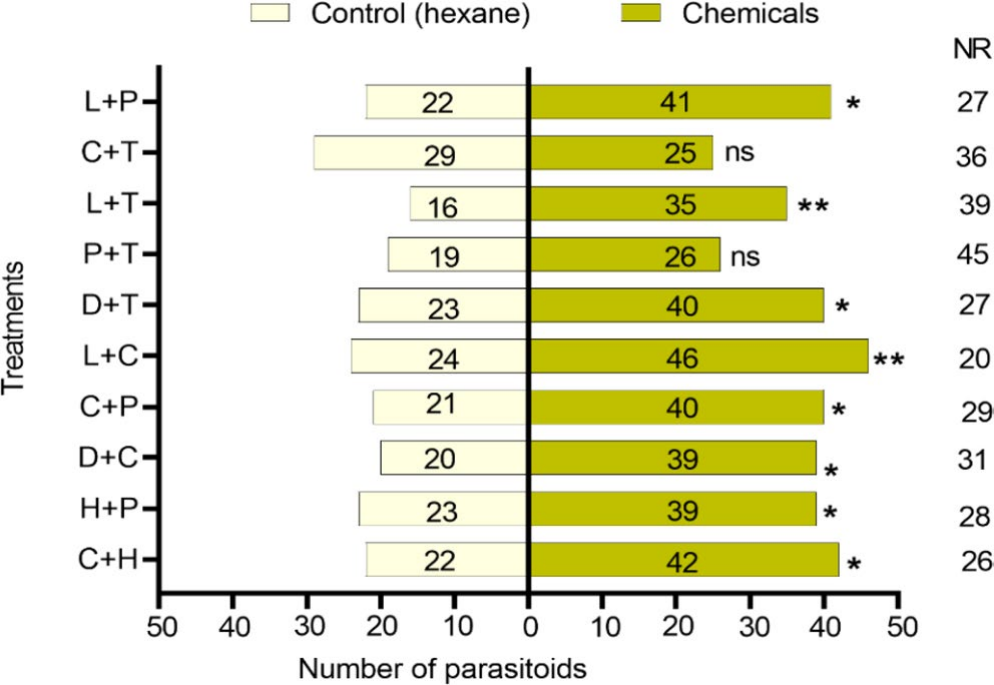
Response of *T. remus* females to binary combination of chemicals. C, Cis‐3‐Hexenyl Acetate; D, Decanal; H, Heneicosane; L, Linalool; P, (+)‐α‐pinene; T, Tetracosane. Bars represent means ± SE; ns represents no significance; an asterisk represents *p < 0.05; **p < 0.01; *χ* ^2^ test.

The best concentration of each compound was considered for the binary and ternary combinations. Binary blends significantly attracted the parasitoid (Figure 8). Cis‐3‐hexenyl acetate + heneicosane (C + H) (*χ* ^2^ = 6.250; df = 1; *p* = 0.012), heneicosane + (+)‐α‐pinene (H + *P*) (*χ* ^2^ = 4.129; df = 1; *p* = 0.042), decanal + cis‐3‐hexenyl acetate (D + C) (*χ* ^2^ = 6.119; df = 1; *p* = 0.013), cis‐3‐hexenyl acetate + (+)‐α‐pinene (C + *P*) (*χ* ^2^ = 5.918; df = 1; *p* = 0.015), cis‐3‐hexenyl acetate + linalool (C + L) (*χ* ^2^ = 6.914; df = 1; *p* = 0.009), decanal + tetracosane (H + T) (*χ* ^2^ = 4.587; df = 1; *p* = 0.032), linalool + tetracosane (L + T) (*χ* ^2^ = 7.078; df = 1; *p* = 0.008), linalool + (+)‐α‐pinene (L + *P*) (*χ* ^2^ = 5.730; df = 1; *p* = 0.017). However, the mixture of *P* + T (*χ* ^2^ = 1.089; df = 1; *p* = 0.297) and the mixture of cis‐3‐hexenyl acetate + tetracosane (C + T) (χ^2^ = 0.296; df = 1; *p =* 0.586) did not show significant attraction.

**FIGURE 9:**
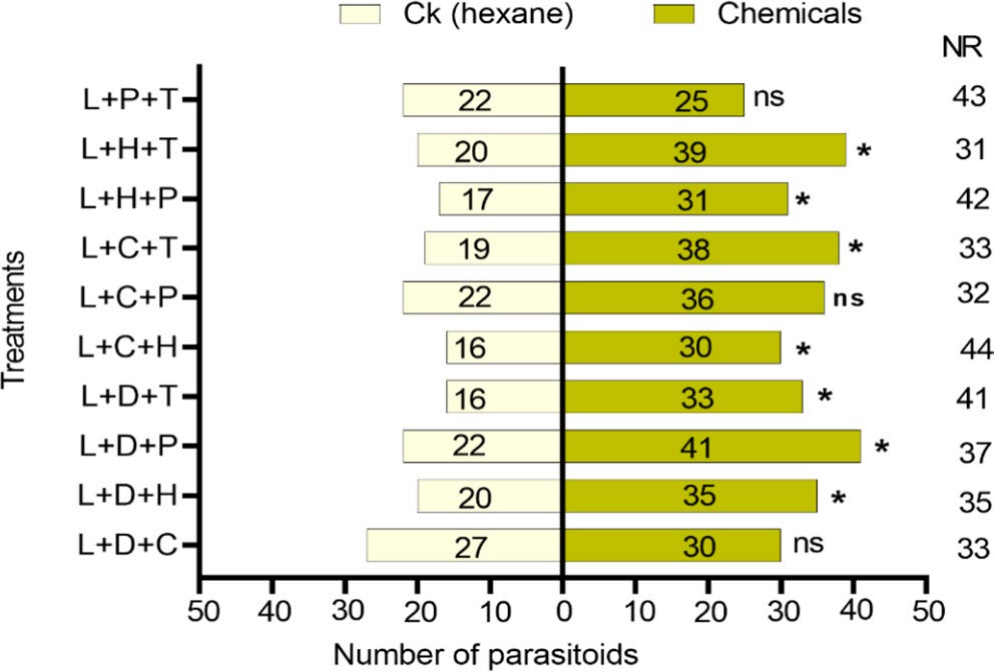
Response of *T. remus* females to combinations of ternary compounds. C, Cis‐3‐Hexenyl Acetate; D, Decanal; H, Heneicosane; L, Linalool; P, (+)‐α‐pinene; T, Tetracosane. Bars represent means ± SE; NR, non‐respondents; ns, no significance, and an asterisk represents *p < 0.05, *χ* ^2^ test.

The combinations of L + D + C (*χ* ^2^ = 0.158; df = 1; *p* = 0.691), L + P + O (*χ* ^2^ = 3.379; df = 1; *p* = 0.066), and L + P + T (*χ* ^2^ = 0.191; df = 1; *p* = 0.662) did not show significant attraction to the parasitoids compared to the control. However, all the other combinations attracted the parasitoid (Figure 9). L + D + P (*χ* ^2^ = 5.730; df = 1; *p* = 0.017), L + D + T (*χ* ^2^ = 5.898; df = 1; *p* = 0.015), L + C + T (*χ* ^2^ = 6.119; df = 1; *p* = 0.013), L + H + T (*χ* ^2^ = 6.333; df = 1; *p* = 0.012), L + D + H (*χ* ^2^ = 4.091; df = 1; *p* = 0.043), L + C + H (*χ* ^2^ = 4.261; df = 1; *p* = 0.039), L + H + P (*χ* ^2^ = 4.083; df = 1; *p* = 0.043). Linalool in particular was very attractive both alone and in combination with other chemicals, except when combined with L + C + P, L + P + T, and L + D + C.

## 4 Discussion

Egg parasitoids utilize both long‐range chemical cues (HIPVs and OIPVs) emitted in response to herbivore activity and short‐range cues derived from the host or its by‐products. Optimizing the composition and concentration of these synthetic volatiles may enhance biological control strategies by selectively attracting natural enemies specific to the target pest species. This study demonstrated that both naïve and experienced *T. remus* were attracted to oviposition‐induced volatiles (OIPVs) and *S. litura* egg volatiles (SEVs), but not to volatiles from tobacco plants with manually introduced eggs (MEIPVs). This suggests that oviposition‐specific elicitors are necessary for plants to release signals that recruit parasitoids or other factors such as the timing of oviposition, host feeding habits, and host abundance (Hilker and Fatouros 2015). Female egg parasitoids, in particular, may be attracted to chemicals from the host, the herbivore host plant, or a combination of both (Greenberg et al. 2023). Among these, OIPVs provide detectable and reliable information about the foraging behavior of egg parasitoids (Milonas et al. 2019; Afentoulis et al. 2021; Salerno et al. 2019; Frati et al. 2017).

The Y‐tube olfactometer is a widely used bioassay for assessing insect behavioral responses to volatile organic compounds. However, several limitations can affect its precision and reliability. Factors such as unbalanced airflow, odor channel contamination, and fixed positioning of the source of stimuli can lead to inconsistencies if not properly controlled (Saitta et al. 2024; Burton et al. 2019; Luquet et al. 2019). In addition, the physiological state of insects, including age, mating status, and previous exposure to odor sources, can substantially influence their behavioral responsiveness (Peri et al. 2016; Pogue et al. 2024). Artificial and simplified test environments are also not ecologically realistic, limiting extrapolation to field conditions (Lin et al. 2022). Additionally, the use of small sample sizes can undermine statistical robustness and lead to biased conclusions (Park and Thompson 2021). In the present study, possible limitations were considered, and measures were taken to minimize experimental bias. To minimize the risk associated with small sample sizes, each chemical was tested with 90 independent replicates. Positional biases were avoided by regularly alternating the olfactometer arms, while the airflow rates and light distribution between the two arms were continuously monitored and maintained under constant conditions.

In this study, naïve parasitoids were significantly attracted to OIPVs collected 36 and 60 h after oviposition but not to volatiles 84 h after oviposition in a Y‐tube olfactometer test. This suggests that parasitoids' response depends on host oviposition timing (Moujahed et al. 2014). Our result aligns with the result reported by Fatouros et al. (2012) that egg parasitoid Trichogramma brassicae was most attracted to volatiles released 24–36 h after oviposition. Experienced parasitoids, on the other hand, responded positively to volatiles emitted 36, 60, and 84 h after oviposition. Prior exposure to *S. litura* eggs increased the attraction of *T. remus* not only to egg volatiles but also to OIPVs. The increased attraction observed in experienced parasitoids likely indicates associative learning, where they associate previous encounters with certain chemicals. Such adaptation can enhance their fitness, reduce search costs, and facilitate rapid dispersal for subsequent attacks. Numerous studies have suggested that egg parasitoids evolve associative learning to search for hosts in complex and dynamic environments (Colazza et al. 2010; Cusumano et al. 2012). Prior experience with host‐plant interactions enables parasitoids to respond more effectively to signals induced by host oviposition (Fatouros et al. 2005).

Our results also demonstrated that volatiles released from *S. litura* eggs (kairomones) effectively attract *T. remus*. Host insects produce distinctive hydrocarbons, fatty acids, and proteins that enhance the searching abilities of predators and parasitoids in their vicinity (Rani and Sandhyarani 2012; Afsheen et al. 2008). These chemicals are essential for host acceptance and influence various insect behaviors, including feeding, mating, and oviposition (Murali‐Baskaran et al. 2020). The response of parasitoids also depends on the feeding habits of the herbivore. For example, specialist parasitoids of generalist herbivores are likely to rely more on cues from the herbivores than on cues from the plants (Morawo and Fadamiro 2019). Similar to our results, Gazit et al. (1996) reported that *T. remus* females were attracted to kairomones emanating from *S. frugiperda* eggs, with the highest attraction observed in females aged 2–4 days. The egg parasitoid, *Trichogramma ostriniae* F., is innately attracted to volatiles from the egg mass of *Ostrinia furnacalis* A. (Lepidoptera: Crambidae) (Yong et al. 2007; Bai et al. 2004). In addition, kairomones have been shown to enhance the searching efficiency and parasitization rates of *Trissolcus japonicus* G. (Hymenoptera: Scelionidae) (Boyle et al. 2020; Richardson et al. 2023) and larval ectoparasitic *Holepyris sylvanidis* J. (Hymenoptera: Bethylidae) (Awater‐Salendo et al. 2023).

The attraction of *T. remus* varied depending on the combinations and concentrations of synthetic volatile compounds. In this study, six volatile compounds (Cis‐3‐hexenyl acetate, linalool, decanal, (+)‐α‐pinene, Heneicosane, and tetracosane) were identified from OIPVs, healthy plants, and *S. litura* eggs. Testing these chemicals in different combinations revealed significant attraction of *T. remus* to both binary and ternary combinations of synthetic compounds, with the binary mixtures attracting the highest number of parasitoids compared to single and ternary compounds. Our results align with those of Takács et al. (1997), who reported that *Apanteles carpatus* (Hymenoptera: Braconidae) was more attracted to a mixture of nonanal and geranyl acetone. In addition, a binary combination of (Z)‐3‐hexenyl acetate and essential oil from the plant *Hemizygia petiolata* (Lamiaceae) in a 1:1 ratio has been shown to attract the parasitoid *Cotesia flavipes* (Ngumbi et al. 2005). The ternary chemical combinations also elicited significant attraction to *T. remus* in the Y‐tube olfactometer. However, fewer parasitoids were attracted to ternary combinations compared to binary ones, suggesting that most olfactory receptor neurons in insects respond to only one or a very limited number of chemical compounds (Meiners et al. 2002; De Bruyne and Baker 2008). A previous study also demonstrated that the parasitoid *T. japonicus* was attracted to a combination of synthetic chemicals, ethyl salicylate, β‐caryophyllene, and decanal in a 1:1:1 ratio (Akotsen‐Mensah et al. 2021). Certain compounds in crude extract or combinations of more than two compounds may have repellent effects or mask the attractiveness of other compounds (D'Alessandro et al. 2009). For example, naïve females of the parasitoid *Microplitis rufiventris* K. (Hymenoptera: Braconidae) have avoided maize HIPVs containing indole but were attracted to mixtures without indole (D'Alessandro et al. 2009). Similarly, another study suggested that isoprene can mask the effect of other chemicals in a blend by interfering with herbaceous plants' ability to attract natural enemies (Loivamäki et al. 2008). These findings emphasize the need for a specific combination of compounds to achieve optimal parasitoid attraction.

*T. remus* was strongly attracted to concentrations of 1 and 10 ng/μL for most chemicals, while 100 ng/μL showed no significant attraction, and 1000 ng/μL resulted in repulsion. This suggests that the parasitoids' highly developed olfactory system is capable of detecting minute chemical fractions in a complex environment. Insect behavior is influenced by specific concentration ratios of single odorants or blends of several components, with each component being detected by specific receptor cells (Tichy et al. 2020; Andersson et al. 2015). Moreover, higher doses of volatile chemicals can repel parasitoids (Ayelo et al. 2021). Our findings of dose sensitivity align with a previous report by D'Alessandro et al. (2009). In contrast to our findings, a higher dose of HIPVs successfully attracted the aphid parasitoid *Aphidius colemani* P. (Storeck et al. 2000). Such variation is expected, as parasitoids frequently exhibit species‐specific responses to varying volatile concentrations. Insects exhibit dose‐dependent responses to volatiles, making it challenging to identify attractant and repellent compounds (Yan and Wang 2006; Ngumbi et al. 2005). The proportion of compounds in a blend and their concentration in the solvent are critical factors influencing insect responses (Revadi et al. 2015). For instance, Bruinsma et al. (2009) reported that volatiles induced by jasmonic acid varied depending on the dose and timing of application. The use of synthetic VOC blends can potentially enhance the effectiveness of biological control. By providing consistent and targeted cues for natural enemies, these blends can optimize parasitoid foraging behavior and improve pest management outcomes in agricultural settings.

## 5 Conclusion

Results confirmed that volatiles from *S. litura* eggs and oviposition‐induced plant volatiles attract *Telenomus remus*. The response of parasitoids was positively influenced by the parasitoids' experience and the timing of host oviposition. Additionally, binary combinations of synthetic volatile compounds, particularly linalool and tetracosane, attracted significantly more parasitoids compared to single or ternary mixtures. These attractive compounds could be used in bait traps in greenhouses and fields to retain parasitoids. The present results contribute valuable insights to the development of environmentally friendly and sustainable pest control strategies for tobacco cutworm, *Spodoptera litura* . Moreover, the results reveal new opportunities to advance research on the chemical ecology of *Telenomus remus*, particularly in the context of its interactions with other lepidopteran pests beyond the extensively studied *S. frugiperda* . However, as these results were obtained under controlled laboratory conditions, they are not sufficient to draw definitive conclusions regarding efficiency in the field. Therefore, further validation under natural field conditions is required to confirm the behavioral relevance and practical applicability of the identified compounds. In addition, molecular studies are required to identify the specific genes involved in the biosynthesis and reception of the behaviorally active semiochemicals.

## Author Contributions

**Ibrahim Osman:** formal analysis (equal), investigation (equal), methodology (equal), writing – original draft (equal). **Zhimin Wang:** methodology (equal), resources (equal), software (equal). **Hongnian Li:** methodology (equal), software (equal), visualization (equal). **Ertao Li:** resources (equal), validation (equal), writing – review and editing (equal). **Honglin Feng:** conceptualization (equal), data curation (equal), writing – review and editing (equal). **Jiao Yin:** funding acquisition (equal), project administration (equal), supervision (equal), writing – review and editing (equal). **Gemei Liang:** data curation (equal), visualization (equal), writing – review and editing (equal). **Zhengling Liu:** conceptualization (equal), data curation (equal), writing – review and editing (equal). **Dekai Ning:** conceptualization (equal), software (equal), writing – review and editing (equal). **Kebin Li:** funding acquisition (equal), project administration (equal), supervision (equal), writing – review and editing (equal). **Yonghui Xie:** project administration (equal), software (equal), writing – review and editing (equal).

## Conflicts of Interest

The authors declare no conflicts of interest.

## Data Availability

The article contains all the data that were created and used in this investigation; the raw data are available at this link https://doi.org/10.6084/m9.figshare.29118221.
